# Supplementary figures and images for: Navigation aid for blind persons by visual-to-auditory sensory substitution: A pilot study
Source: PLoS One. 2020 Aug 20;15(8):e0237344. doi: 10.1371/journal.pone.0237344 (PMC7446825; doi:10.1371/journal.pone.0237344)

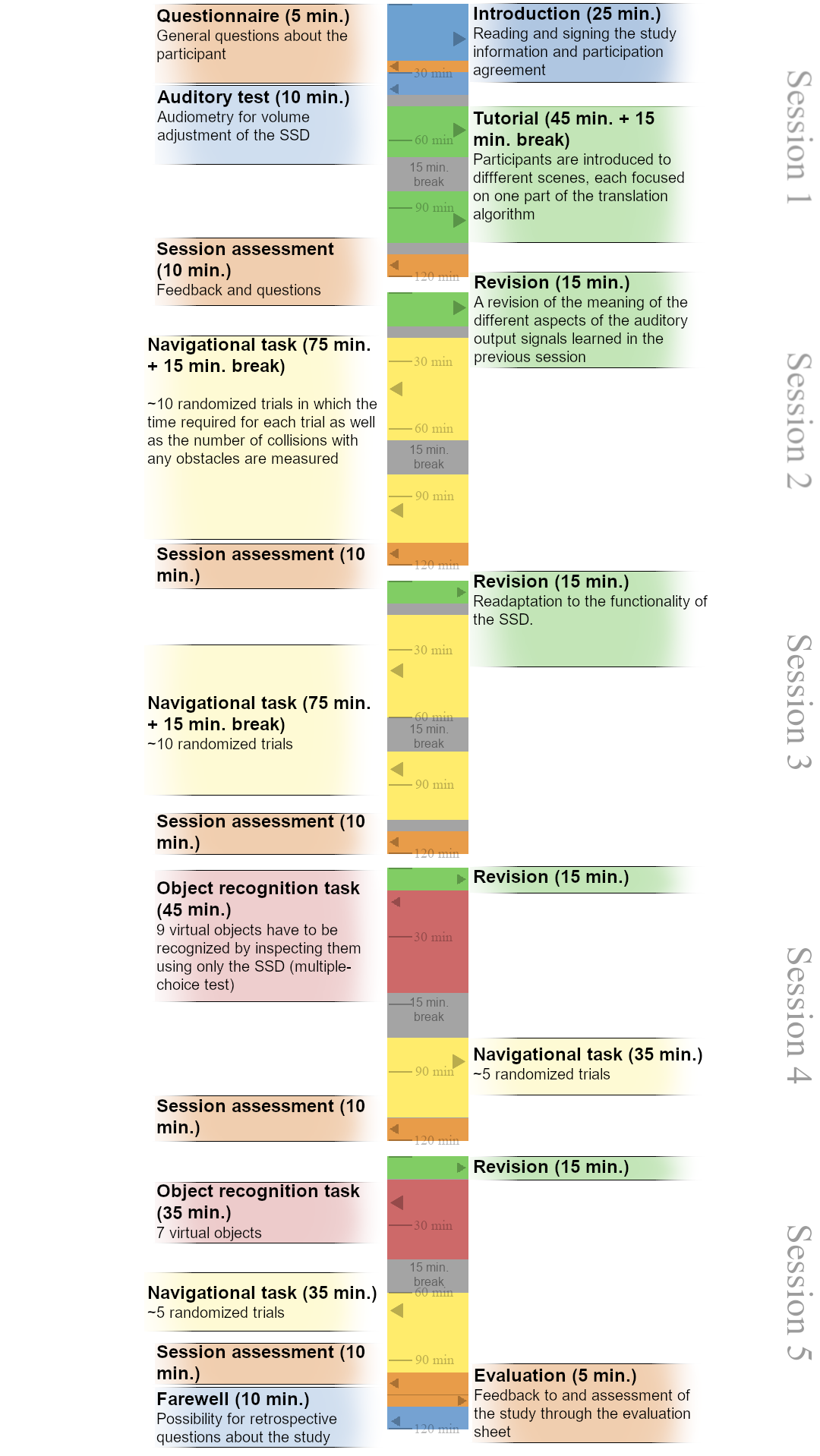

Supplement: S1 Fig — The timetable for the five sessions of the study. (TIF) [file pone.0237344.s001.tif]

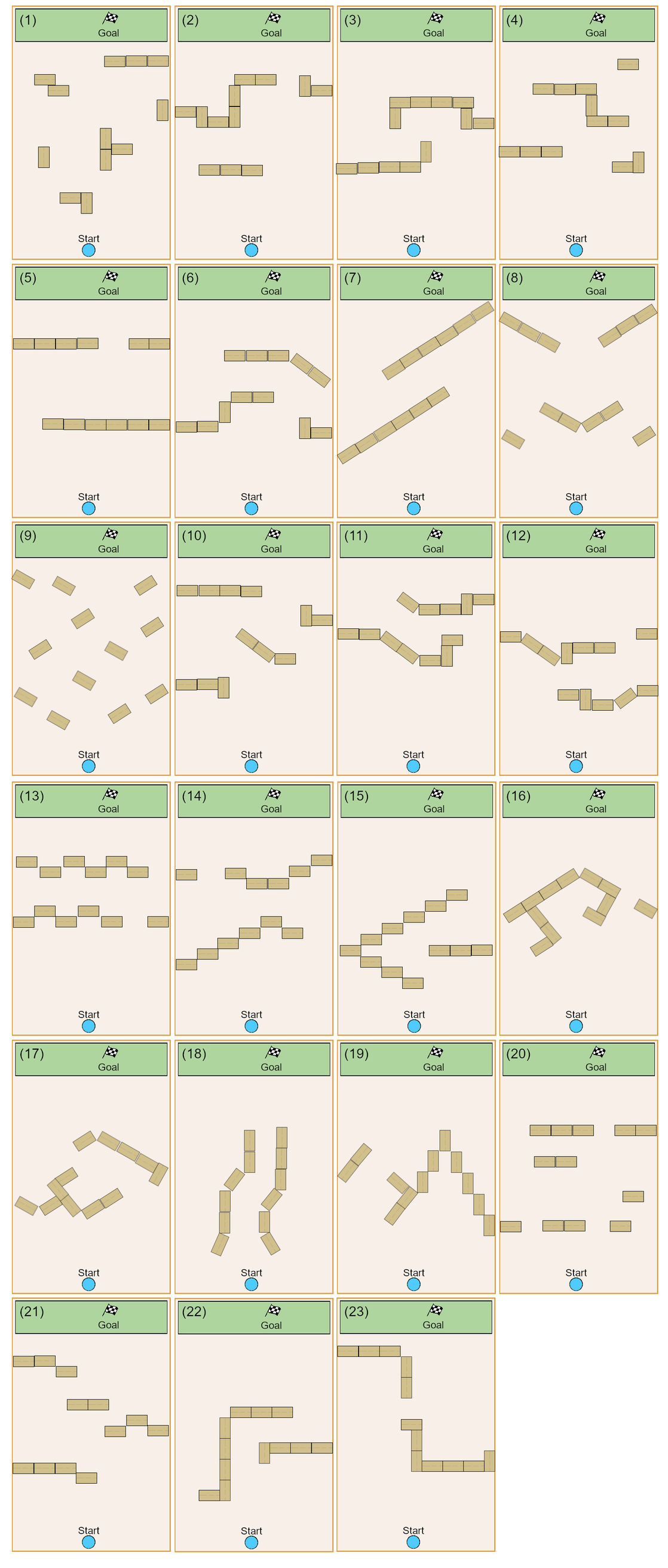

Supplement: S2 Fig — The 23 layouts of the obstacle courses used for the navigation task. (TIF) [file pone.0237344.s002.tif]
